# Coach Education and Positive Youth Development as a Means of Improving Australian Sport

**DOI:** 10.3389/fspor.2020.591633

**Published:** 2020-11-20

**Authors:** Jaimee E. Bateman, Geoff P. Lovell, Karena J. Burke, Michele Lastella

**Affiliations:** ^1^School of Health, Medical and Applied Sciences, Central Queensland University, Rockhampton, QLD, Australia; ^2^Department of Sport, Hartpury University, Hartpury, United Kingdom; ^3^School of Health and Behavioural Sciences, University of the Sunshine Coast, Sunshine Coast, QLD, Australia; ^4^School of Health, Medical and Applied Sciences, Appleton Institute for Behavioural Science, Central Queensland University, Adelaide, SA, Australia

**Keywords:** positive youth development, coaching education, Australian youth sport, youth athlete development, youth sport coaches

## Introduction

Sport plays a large role in Australian culture (Light, 2010) with over 90% of Australians involved in playing or watching sport (Australian Sports Commission, 2017). However, retention of Australian athletes is at risk (Australian Sports Commission, 2017). Protecting Australian sport from decreased participation across all ages is vital to maintaining the intergenerational cycle of Australians playing sport (Australian Sports Commission, 2017). Sport is important to Australians' mental and physical health and contributes substantially to the economy (Australian Sports Commission, 2017). To address the prospect of declining participation, the Australian Sports Commission (2017) has declared a focus toward increasing youth athlete participation by the year 2036.

The objective of this paper is to suggest that Australian sport coaches, are vital in sustaining Australian youth sport participation (Duda, 1996; Cote and Mallett, 2012; Vella et al., 2013). Through coach education, coaches have the potential to enhance motivational climates that foster positive youth development (Duda, 1996; Falcao et al., 2012; Bailey et al., 2013; Santos et al., 2017). Positive youth development through sport occurs when young athletes obtain personal, physical and social skills from playing sport, that can be transferred to other areas of their lives; improving their present and future well-being and societal contributions (Holt et al., 2016).

Positive youth development through sport has been shown to increase participant retention (Smith and Smoll, 1997; Cote et al., 2010), due to enhancing athletes' general well-being (Roth and Brooks-Gunn, 2003; Falcao et al., 2012) and consequently boosting their enjoyment of sport. However, research on positive youth development in Australian sport is lacking (Light, 2010; Gould, 2016). More Australian studies are required to investigate if current coach education is adequate in providing coaches with knowledge and skills to foster positive youth development. Thus, this paper will conclude with research recommendations aimed at advancing our understanding of the effectiveness of Australian coaches and Australian coach education in promoting positive youth development through sport.

## Motivational Requirements for Positive Youth Development

*Positive youth development* occurs when children's values, beliefs and life-skills are proactively strengthened to enable maturation into well-balanced, optimal-functioning individuals (Gould and Carson, 2008). For positive youth development to occur, attention must be given to the motivational climate surrounding young people (Dweck, 1986; Bailey et al., 2013). The *motivational climate* is created through the way that influential adults define success; consequently, shaping how children interpret their efforts toward achieving goals (Dweck, 1986).

Two types of motivational climates have been identified; mastery and performance (Duda, 1996). In *mastery-orientated* environments, rather than emphasizing goal attainment, success is interpreted as self-improvement obtained when working toward one's goals (Duda, 1996). Focusing on the enjoyment and satisfaction of progress, especially through challenges and overcoming failures, teaches individuals social-emotional skills such as self-determination, work ethic and citizenship (Dweck, 1986). In contrast, *performance-orientated* environments can be detrimental to personal growth as they emphasize social comparisons and superior outcomes over others, with goal attainment the definition of success (Dweck, 1986). Performance-orientated individuals often seek easy pathways to achievement so as to avoid failure and social judgements, but in the process prevent themselves from developing social-emotional skills and satisfaction in the progress made toward goal attainment (Duda, 1996).

## Fostering Positive Youth Development Through Sport

Sport is often considered training for real-life (Petitpas et al., 2005) and ideal for enhancing youth's positive development (Vella et al., 2011; Camire, 2015). *Positive youth development through sport* goes beyond building athletes' sport specific abilities, and aims to cultivate their psychological, social, emotional, physical and intellectual skills (Roth and Brooks-Gunn, 2003; Cote et al., 2010; Santos et al., 2017) that can be utilized in sport and life (Gould and Carson, 2008; Falcao et al., 2012). Mastery-orientated contexts that de-emphasize the outcome of winning and prioritize athletes' self-improvement in abilities and sportsmanship (Duda, 1996; Roth and Brooks-Gunn, 2003; Bailey et al., 2013), have been reported as necessary for positive youth development through sport (Cote and Mallett, 2012). Mastery-orientated sports provide athletes with opportunities to experience challenges, independence and cooperation; all while athletes' efforts are supported and encouraged (Dweck, 1986; Ames and Archer, 1988; Bailey et al., 2013). Athletes who feel safe and supported in taking risks toward reaching goals, regardless of the outcome, are more likely to engage in activities that foster their cognitive and social-emotional skills (Duda, 1996; Falcao et al., 2012; Vella et al., 2013). The support, enjoyment and positive development experienced in mastery-orientated sports, as well as increased overall well-being (Roth and Brooks-Gunn, 2003; Camire and Trudel, 2014), reinforces athletes' desire to remain playing sport (Ames and Archer, 1988; Cote and Mallett, 2012; Bailey et al., 2013).

## The Importance of Coaches

In the context of sport, youth coaches are considered teachers and leaders (Feltz et al., 1999). Parents, athletes and sporting organizations entrust coaches to help athletes develop, both in and out of the sporting arena (Camire, 2015; Strachan et al., 2016). As the primary influences on the sporting climate (Ames and Archer, 1988; Duda, 1996; Bailey et al., 2013), coaches are pivotal in fostering positive youth development through sport (Cote et al., 2010; Camire et al., 2012; Vella et al., 2013). When coaches deliberately create mastery-orientated environments that aim to develop athletes beyond sporting skills and tactics, positive youth development becomes more likely (Holt et al., 2017). Creating a mastery-orientated environment conducive to positive youth development, requires coaches emphasize the goal of sport as being to learn from mistakes while enjoying working hard for personal improvement (Duda, 1996). Coaches should focus on athletes' development rather than performance, with athletes evaluated against themselves, not others (Ames and Archer, 1988). It is vital that coaches make all athletes feel important and acknowledged (Dweck, 1986). Coaches can further encourage athletes' positive development by incorporating deliberate lessons into their programs, designed to teach life-skills (Gould and Carson, 2008; Holt et al., 2017) such as emotional control (Falcao et al., 2012), cultural competence, personal responsibility, and interpersonal skills (Light, 2010; Camire et al., 2012). Finally, improving youth athletes' positive development can be achieved through coaching behaviors such as role modeling, fostering strong relationships, using empathetic communication (Smith and Smoll, 1997), and positive reinforcement (Gould et al., 1989).

## Problems With Positive Youth Development Through Sport

Athlete development through sport is, unfortunately, not always positive (Shields and Bredemeier, 2010). Just as coaches shape mastery-orientated environments, they also influence performance-orientated athletes (Duda, 1996), possibly due to cultural norms of winning at all costs (Cote and Mallett, 2012). Currently, youth sports in Australia are predominantly performance-driven (Cote and Mallett, 2012; Agnew et al., 2016). Athletes who participate in performance-driven sports are more likely to develop negatively (Cote et al., 2010) and experience increased adversity in (Camire et al., 2012) and out of sport (Gould and Carson, 2008). In their report to the Australian Sports Commission, Cote and Mallett (2012) suggested that athlete attrition in Australian youth sports may be attributed to the performance-driven sporting culture and its lack of emphasis on positive youth development.

Most coaches understand that they can inspire positive youth development (Gould et al., 2006; Vella et al., 2011, 2013; Santos et al., 2017); however, preliminary data suggests they do not fully understand why (Bean and Forneris, 2017). Many coaches do not actively foster positive youth development in athletes, believing positive development occurs through participation alone (Bean and Forneris, 2017). Other coaches take a reactive approach, only addressing personal development if problems occur (Zakrajsek and Zizzi, 2008). Coaches who do aim to incorporate positive youth development, often use limited positive youth development methods (Gould et al., 1989) or inadvertently employ approaches that negatively impact youth development (Dweck, 1986; McCallister et al., 2000).

A lack of education on positive youth development in sport (Erickson et al., 2008; Harwood, 2008; Santos et al., 2017) may attribute to coaching mistakes and limitations (Lerner et al., 2005; Strachan et al., 2016) that negatively influence athletes (Petitpas et al., 2005; Cote et al., 2010; Vella et al., 2013). Positive youth development coach education programs help coaches develop confidence (Falcao et al., 2012; Santos et al., 2017), self-awareness (Smith and Smoll, 1997) and knowledge (Vella et al., 2013) in fostering positive development in athletes. Despite the advantages of educating coaches on positive youth development, most compulsory coach education courses focus primarily on sporting skills and tactics (Santos et al., 2017). Little importance is given in coach education to the holistic development of athletes, especially at the community level (Wiersma and Sherman, 2005).

## The Need for Mandatory Positive Youth Development Coach Education

International research provides a strong argument for the mandatory inclusion of positive youth development components in Australian coach education (Erickson et al., 2008; Harwood, 2008; Falcao et al., 2012; Strachan et al., 2016). For example, without being part of mandatory education, positive youth development in sport is devalued (Harwood, 2008; Falcao et al., 2012; Strachan et al., 2016) and difficult for coaches to source (Erickson et al., 2008; Strachan et al., 2016). Positive youth development education programs are available online, however most coaches are unaware of their existence (Nash and Sproule, 2012).

Introducing mandatory positive youth development education would ensure all Australian coaches are aware of the importance of positive youth development in sport (Harwood, 2008) and equipped with the knowledge, confidence and skills to foster positive development in larger populations of athletes (Falcao et al., 2012). Larger numbers of positively developed athletes will increase sport participation rates (Cote and Mallett, 2012), athletes' longevity in sport (Camire et al., 2012), individual contributions to society (Lerner et al., 2005), sporting organisations' sustainability, and the Australian economy (Australian Sports Commission, 2017). Therefore, coach education that includes positive youth development education, not just sport specific information, will help coaches create mastery-orientated sporting environments that enable youth to develop into healthy, functioning adults with less destructive behaviors, improved civic engagement (Lerner et al., 2005) and sustained sports participation (Duda, 1996). Successful integration of positive youth development coach education into Australian youth sport will require the collaboration of governing sporting bodies, policymakers, education providers, coaches, sporting clubs, and parents (Camire, 2015; Australian Sports Commission, 2017).

## Shortfalls in Australian Research

Positive youth development has been studied from various theoretical perspectives including motivation theory, self-determination theory, social learning theory and ecological systems theory (Lerner et al., 2005; Weiss, 2016), however few real-world applications have resulted (Weiss, 2016). To date there has been a lack of large-scale research on positive youth development in Australian sport to support claims that increasing coach education on positive youth development will actually benefit Australian sport (Light, 2010; Vella et al., 2011). There is currently little research within the Australian context to answer the following pertinent questions:

- Is there concordance between what Australian coaches should know and practice regarding positive youth development in sport and what they actually do understand and apply (Bailey et al., 2013)?
- Do Australian coaches have efficient access to quality positive youth development coach education (Nash and Sproule, 2012; Bailey et al., 2013; Pope et al., 2015) that has been empirically tested for Australian youth sporting populations (Conroy and Coatsworth, 2006; Gould, 2016)?
- What opinions and knowledge do Australian coaches and parents have about coach education and positive youth development in sport (Wiersma and Sherman, 2005; Vargas-Tonsing, 2007; Bailey et al., 2013; Camire and Trudel, 2014; Newman et al., 2016; Santos et al., 2017)?
- What cultural impacts and individual beliefs influence positive youth development in Australian sport (Bailey et al., 2013)?

## Conclusion

The objective of this paper was to stress the important role of coach education in providing coaches with information on creating mastery-orientated environments that foster positive youth development. Past research indicates that positive youth development through sport benefits individual athletes, coaches, families, sporting organizations, and communities. The diverse benefits of positive youth development through sport, suggest that enhancing positive youth development in Australian sport through improved, mandatory coach education may have profound consequences, including sustained youth athlete participation. However, more research is needed to determine if international findings are relevant for Australian sport and to further understand how positive youth development can be fostered through Australian sport to benefit everyone involved.

## Author Contributions

JB conceived the original idea for the commentary and drafted the article. ML oversaw the project and offered advice on direction for the paper. ML, GL, and KB critically revised the paper and contributed ideas to the final article. ML gave final approval. All authors contributed to the article and approved the submitted version.

## Conflict of Interest

The authors declare that the research was conducted in the absence of any commercial or financial relationships that could be construed as a potential conflict of interest.
